# Anti‐Inflammatory and Antinociceptive Potential of Phaeocaulisin H From *Curcuma wilcockii* Rhizomes (*Zingiberaceae*) in In Vitro, In Vivo, and In Silico Studies

**DOI:** 10.1155/adpp/2384820

**Published:** 2026-11-09

**Authors:** A. B. M. Ashraful, Md. Sabbir Hossain, Farhad Hossain, Joy Sarker, Md. Najem Uddin, Aziz Abdur Rahman, Md. Golam Sadik, Mamunur Rashid, A. H. M. Khurshid Alam

**Affiliations:** ^1^ Department of Pharmacy University of Rajshahi Rajshahi 6205 Bangladesh ru.ac.bd; ^2^ Department of Pharmacy Pabna University of Science and Technology Pabna 6600 Bangladesh pust.ac.bd; ^3^ Department of Pharmacy State University of Bangladesh Dhaka 1461 Bangladesh sub.edu.bd; ^4^ Bangladesh Council of Scientific and Industrial Research (BCSIR) Dhanmondi Dhaka 1205 Bangladesh bcsir.gov.bd

**Keywords:** anti-inflammatory activity, antinociceptive activity, *Curcuma wilcockii*, phaeocaulisin H, sesquiterpene

## Abstract

*Curcuma wilcockii*, a lesser‐explored member of the Zingiberaceae family, has been traditionally used in folk medicine, but its phytochemical compositions and pharmacological activities remain poorly investigated. This study evaluates the anti‐inflammatory and antinociceptive potential of phaeocaulisin H (PN‐H), a guaiane‐type sesquiterpene newly isolated from *C. wilcockii* rhizomes, using in vitro, in vivo, and in silico approaches. In vitro activity was assessed using heat‐induced RBC hemolysis and egg albumin denaturation assays. In vivo anti‐inflammatory effects were evaluated using carrageenan‐induced paw edema, and antinociceptive activity was assessed using acetic acid‐induced writhing and formalin‐induced licking tests. The COX‐2 docking was performed using PyRx and Biovia Discovery Studio, and pharmacokinetic properties were evaluated using SwissADME. PN‐H was isolated and characterized by LC‐MS, ^1^H, ^13^C, and 2D‐NMR. It significantly reduced hemolysis (93.55%) and protein denaturation (80.86%) at a concentration of 25 μg/mL. It inhibited paw edema by 32.61% and 50% at the doses of 10 and 20 mg/kg body weight (b.w.), respectively. In the writhing test, PN‐H showed 50.93% and 79.13% (*p* < 0.0001) inhibition, whereas in the formalin test, it reduced early‐phase licking by 56.77% and 70.32% and late‐phase licking by 52.78% and 77.78% (*p* < 0.001). Docking studies revealed a favorable predicted interaction with COX‐2, while ADME analysis predicted acceptable drug‐likeness. To the best of our knowledge, this study reports for the first time the isolation of PN‐H from *C. wilcockii* and provides evidence of its anti‐inflammatory and antinociceptive activities. The findings support further molecular and pharmacological investigation of PN‐H as a potential anti‐inflammatory candidate.

## 1. Introduction

Inflammation is a complex and tightly regulated biological response to harmful stimuli, tissue damage, or microbial invasion. It serves as a fundamental component of the innate immune system [1]. Inflammation can be acute or chronic. Acute inflammation plays a pivotal role in restoring tissue integrity and maintaining physiological homeostasis. In contrast, chronic or dysregulated persistence of acute response is increasingly recognized as a major contributor to the pathophysiology of numerous long‐term and progressive disorders such as rheumatoid arthritis, cardiovascular diseases, neurodegenerative conditions, and various forms of cancer [2]. The widespread incidence and clinical burden of these inflammation‐driven diseases highlights a critical need for the development of effective, selective, and safe anti‐inflammatory therapeutics. In response, contemporary biomedical research increasingly focuses on discovering novel bioactive compounds that can regulate critical inflammatory pathways, suppress abnormal immune activation, and ultimately restore immune and tissue function [3]. Natural products from plants, especially secondary metabolites, have been a rich source of drugs because of their wide structural diversity and strong biological activities [4]. Among these metabolites, sesquiterpenes are a group of terpenoids composed of three isoprene units forming a 15‐carbon backbone. They are valued for their broad spectrum of pharmacological properties, particularly anti‐inflammatory activities [5]. Within this versatile group, guaiane‐type sesquiterpenes are of particular interest owing to their distinctive molecular frameworks and pronounced therapeutic potential. Their unique ring systems and stereochemical complexity confer significant bioactivity, highlighting their potential as lead compounds for new therapeutics [6]. Among the diverse phytochemicals reported from *Curcuma* species, guaiane‐type sesquiterpenes of the phaeocaulisin series have attracted attention because of their distinctive structures and biological activities. Phaeocaulisins A–J were originally isolated from the rhizomes of *Curcuma phaeocaulis*, and several compounds from this series were evaluated for inhibitory effects on LPS‐induced nitric oxide production in RAW 264.7 macrophages [7]. These findings suggest that phaeocaulisin‐type sesquiterpenes may possess anti‐inflammatory potential. However, information regarding the occurrence and pharmacological properties of individual phaeocaulisin derivatives in other *Curcuma* species remains comparatively limited. Therefore, further investigation of these compounds in less‐studied *Curcuma* species may provide additional insight into their chemical distribution and pharmacological potential.

Belonging to the Zingiberaceae family, *Curcuma* species have a long history of use in South and Southeast Asian traditional medicine for the management of inflammation and infectious disorders. Well‐known species such as *Curcuma longa* (turmeric) and *Curcuma zedoaria* have been widely studied and are recognized for their bioactive compounds, such as curcumin, which have multiple therapeutic effects [8]. In contrast, *Curcuma wilcockii* remains comparatively underexplored in phytochemical and pharmacological research. Therefore, investigating this lesser‐known species may expand our understanding of the chemical diversity and pharmacological potential of the genus *Curcuma*.

Previous studies on guaiane‐type sesquiterpenes isolated from *Curcuma* and related medicinal plants have demonstrated their ability to suppress key inflammatory mediators, including pro‐inflammatory cytokines such as TNF‐α (tumor necrosis factor‐alpha) and IL‐6 (interleukin‐6), as well as inducible enzymes including COX‐2 (cyclooxygenase‐2) and iNOS (inducible nitric oxide synthase), through modulation of important intracellular signaling pathways [9]. In addition, several compounds belonging to this structural class have shown anti‐inflammatory efficacy and acceptable tolerability in experimental animal models, supporting their pharmacological relevance under physiologically relevant conditions [10]. Complementary computational investigations, including molecular docking and pharmacokinetic predictions such as ADMET (absorption, distribution, metabolism, excretion, and toxicity) profiling and pharmacophore analysis, have further suggested favorable interactions of these sesquiterpenes with inflammatory targets, indicating promising drug‐likeness properties [11]. Collectively, these findings highlight the anti‐inflammatory potential of guaiane‐type sesquiterpenes and provide a scientific basis for evaluating PN‐H as a candidate compound in the present study.

COX‐2 plays a central role in inflammatory processes by catalyzing the conversion of arachidonic acid into prostaglandins, which are key mediators responsible for pain, edema, and inflammatory responses [12]. Selective inhibition of COX‐2 is therefore considered an effective therapeutic strategy for controlling inflammation while minimizing gastrointestinal side effects associated with non‐selective NSAIDs. Several natural products, particularly terpenoids and sesquiterpenes, have been reported to modulate inflammatory pathways through inhibition of COX‐2 expression or activity [13]. Guaiane‐type sesquiterpenes, characterized by their bicyclic carbon skeleton and hydrophobic framework, can interact with enzyme‐binding pockets through hydrophobic and hydrogen‐bond interactions [14]. These structural characteristics suggest that such compounds may act as potential COX‐2 inhibitors. Therefore, COX‐2 was selected as the primary molecular target in this study to explore the potential anti‐inflammatory mechanism of PN‐H through molecular docking analysis.

Therefore, the present study aimed to investigate the anti‐inflammatory and antinociceptive potential of PN‐H isolated from *C. wilcockii* using integrated in vitro, in vivo, and in silico approaches. In particular, molecular docking analysis was performed to evaluate the interaction of PN‐H with the COX‐2 enzyme, providing mechanistic insight into its possible anti‐inflammatory activity.

## 2. Materials and Methods

### 2.1. Reagents

All chemicals and reagents used in this study were obtained from established commercial suppliers. Methanol, chloroform, and n‐Hexane were procured from DAEJUNK Company Limited, Busan, Korea. Formalin and acetic acid were sourced from Merck & Co. (India). Diclofenac sodium (DS), acetylsalicylic acid, and carrageenan were purchased from Sigma‐Aldrich (USA).

### 2.2. Collection, Extraction, and Fractionation

Fresh Rhizomes of *C. wilcockii* were collected from Moulvibazar, Bangladesh, in December 2023 (Figure 1). The plant material was authenticated by a specialist at the Mirpur National Herbarium, Dhaka, Bangladesh, and deposited under voucher number 104531. Approximately 3 kg of fresh rhizomes were obtained for the study and subsequently air‐dried at room temperature for 1 month. Following drying, the rhizomes were chopped and ground into a coarse powder using an electric grinding apparatus (NOVENA Grinding Mixer, India), yielding 630 g of pulverized material [15]. Exhaustive extraction was performed using a Soxhlet apparatus (BÜCHI Labortechnik GmbH, Essen, Germany) with methanol as the solvent. A rotary evaporator (Yamato Scientific Co., Ltd., Chuo‐ku, Tokyo, Japan) was used to evaporate the solvent at 40°C under reduced pressure, yielding 40 g of concentrated methanolic crude extract. The Kupchan partitioning method was applied to separate the crude extract into NHF, CHF, ethyl acetate fraction (EAF), CME, and AQF using solvents of increasing polarity, as shown in the flowchart in Supporting Figure S1 [16].

**FIGURE 1:**
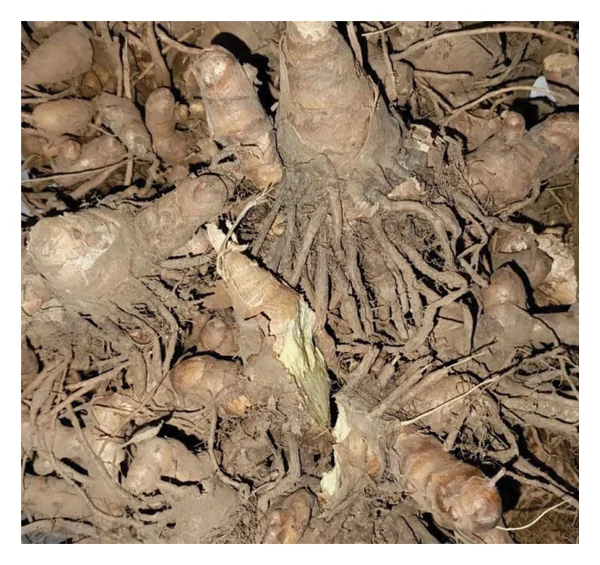
Rhizome of *Curcuma wilcockii*.

### 2.3. Isolation and Characterization of Compound

A 250 mg of the EAF obtained from *C. wilcockii* rhizomes was subjected to silica gel column chromatography using solvent systems of increasing polarity. A total of 207 eluates were collected and grouped into eight major fractions (Fr‐1 to Fr‐8) based on their identical thin‐layer chromatography (TLC) profiles (Supporting Table S1). All fractions were analyzed by TLC using a chloroform: methanol (9.5:0.5, v/v) solvent system. The fraction 5 (Fr‐5, 25 mg) exhibited a single distinct TLC spot, indicating the presence of a purified compound, and was designated as CW‐EAF (*Curcuma wilcockii* ethyl acetate fraction). Structural elucidation of the isolated compound was performed using nuclear magnetic resonance (NMR) spectroscopy on a Bruker DPX‐400 spectrometer operating at 400 MHz for 1H NMR and 100 MHz for 13C NMR (Figure 2). The structure was established through detailed analysis of the spectral data and comparison with previously published reference values (Supporting Table S2) [7].

**FIGURE 2:**
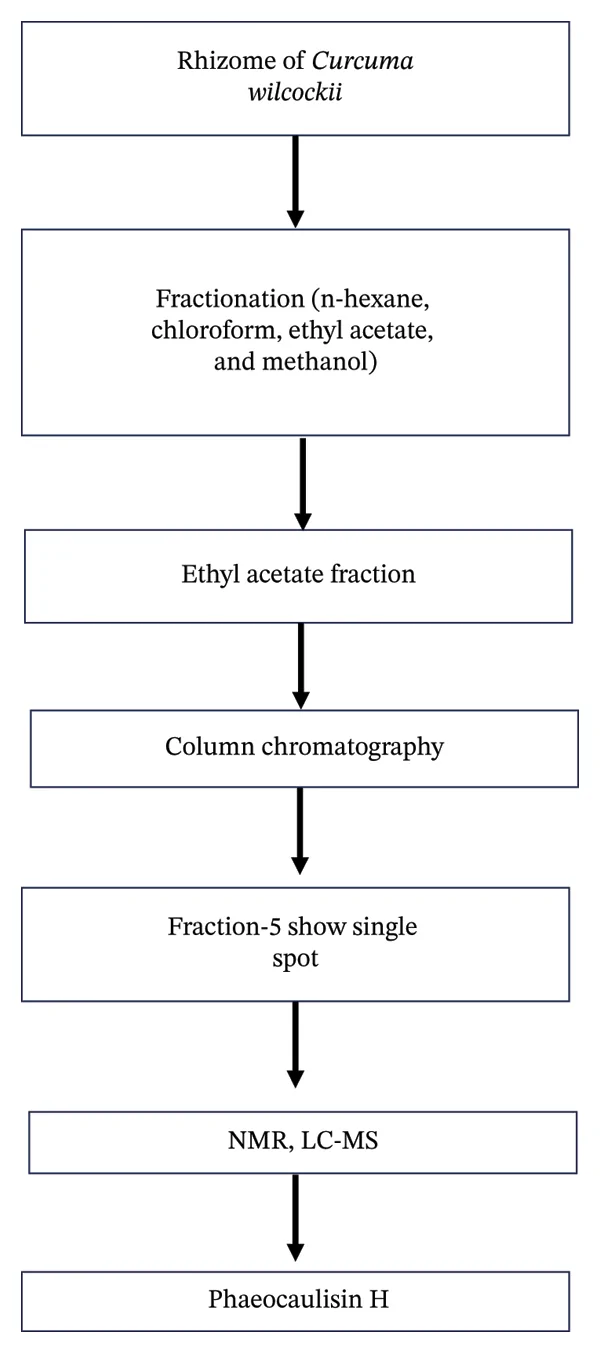
Schematic flowchart of the isolation of the PN‐H.

### 2.4. In Vitro Anti‐Inflammatory Activity

#### 2.4.1. Heat‐Induced RBC Hemolysis

The heat‐induced red blood cell (HRBC) membrane stabilization assay for PN‐H was conducted following a previously established protocol with slight modifications [17, 18]. Blood samples were collected from a healthy volunteer who was not taking non‐steroidal anti‐inflammatory drugs (NSAIDs). Next, they were centrifuged for 10 min at 3000 rpm. After three washes, the washed RBCs were suspended in a 10% RBC suspension and stored at 4°C.

A volume of 5 mL isotonic buffer containing varying concentrations (3.125, 6.25, 12.5, 25, and 50 μg/mL) of PN‐H was added to duplicate sets of tubes. An identical volume of the control solution was put into another tube. A suspension of 50 μL of RBCs was added to each tube, and the tubes were mixed by inversion. One set of tubes was incubated in a water bath at 54°C for 20 min, while the corresponding duplicates were maintained in an ice bath at 0°C–5°C. After incubation, all samples were centrifuged at 500 rpm for 5 min. The absorbance of collected supernatants was measured at 560 nm using a UV–visible spectrophotometer.

Acetylsalicylic acid (ASA, 200 μg/mL) was used as the standard. This process was carried out three times at every concentration. The percentage inhibition of hemolysis was calculated using the following formula:

(1) % inhibition of hemolysis=1−OD2−OD1OD3−OD1×100,

where *O* *D*_1_ = Test sample unheated, *O* *D*_2_ = Test sample heated, and *O* *D*_3_ = Control sample heated.

#### 2.4.2. Protein Denaturation Using Egg Albumin

A 5 mL reaction mixture was prepared, comprising egg albumin (0.2 mL), phosphate‐buffered saline (2.8 mL PBS, pH 6.4), and PN‐H (2 mL) at different concentrations (3.125, 6.25, 12.5, 25, and 50 μg/mL). After incubation of the mixture at 37°C ± 2°C for 15 min, it was heated at 70°C for 5 min. After cooling to room temperature, the absorbance was measured at 660 nm using a UV–visible spectrophotometer. DS was employed as the standard at different concentrations (3.125, 6.25, 12.5, 25, and 50 μg/mL) [17]. This process was carried out three times at every concentration. The following equation was employed to determine the % inhibition of protein denaturation:

(2) % inhibition of protein denaturation=Absorption of control−Absorption of standard/sampleAbsorption of control group×100.

### 2.5. Selection and Grouping of Animals

Swiss albino mice weighing 25–30 g at 4 weeks were obtained from the animal house located at the University of Rajshahi, Bangladesh. The mice were housed in propylene cages under controlled conditions (temperature: 25°C ± 2°C; relative humidity: 55%–65%) with a 12‐h light/dark cycle. All mice received an ad libitum standard diet formulated by ICDDR, B, and water. In the 12 h leading up to studies, both food and water were removed to ensure hydration levels remained consistent [19] as follows:

 Group I: Control group
 Group II: Standard (Diclofenac sodium, 10 mg/kg b.w.)
 Group III: Low dose of PN‐H (10 mg/kg b.w.)
 Group IV: High dose of PN‐H (20 mg/kg b.w.) [20]

The animal experiments were conducted and reported in accordance with the ARRIVE 2.0 guidelines [21]. Animals were randomly allocated to the respective experimental groups, and investigators involved in outcome assessment and data analysis were blinded to group allocation. No animals were excluded from the study or subsequent analyses. The sample size (*n* = 5 per group) was determined based on previously published studies using comparable experimental models and outcome measures.

### 2.6. In Vivo Anti‐Inflammatory Activity

#### 2.6.1. Carrageenan‐Induced Paw Edema

The anti‐inflammatory effect of PN‐H was assessed using the carrageenan‐induced paw edema model in mice. Four groups of male mice (*n* = 5 per group) were used for the carrageenan‐induced paw edema assay. The sample size of five animals per group was selected based on previously published studies using similar experimental models [19]. A formal a priori statistical power calculation was not performed. Acute inflammation was induced by subplantar injection of 0.1 mL of 1% (w/v) carrageenan solution into the right hind paw of each mouse 1 h after oral administration of the respective treatment. The control group was administered normal saline (10 mL/kg b.w.) orally, while the standard group (Group II) was administered DS at a dose of 10 mg/kg b.w. The test groups (Group III and IV) were treated with PN‐H at doses of 10 mg/kg and 20 mg/kg b.w., respectively. Paw circumference was measured at 0, 30, 60, 120, 180, and 240 min (0–4 h) using Vernier calipers. Paw circumference was selected as a practical and reproducible measure of carrageenan‐induced paw edema, with measurements consistently taken at the same anatomical site for all animals throughout the experiment. The percentage inhibition of paw edema was calculated using the following formula:

(3) Inhibition of paw edema %=Thickness of paw in control group−Thickness of paw in treatment groupThickness of paw in control group×100.

#### 2.6.2. Acetic Acid–Induced Abdominal Writhing Test

The male animals were divided into four groups (*n* = 5 per group). Group I served as the control and received only the vehicle (10 mL/kg, b.w. orally). Groups III and IV received PN‐H at 10 and 20 mg/kg b.w. orally, respectively, while Group II was administered the DS at 10 mg/kg b.w. orally. After 30 min, all groups experienced an intraperitoneal injection of a 1% acetic acid solution intended to induce writhing. The number of abdominal writhes was recorded over 20 min following the injection [22, 23]. The writhe counts for each treatment cohort were contrasted with the controls’ results, allowing calculation of the percent inhibition observed in the different groupings.

(4) % inhibition of abdominal writhing= Nm−NcNm ×100,

where *N*_*m*_ is the number of writhes in the control group and *N*_*c*_ is the number of writhes in the treated group.

#### 2.6.3. Formalin‐Induced Licking Test

The formalin‐induced licking test was conducted according to the method described by Al‐Harrasi [24]. Twenty male mice were randomly divided into four groups (*n* = 5 per group). Group I received the vehicle only and served as the control group. Group II was given DS (10 mg/kg b.w. orally) and served as the standard group. Groups III and IV were administered PN‐H at doses of 10 and 20 mg/kg b.w., respectively, via oral gavage. Thirty minutes later, all mice were injected beneath the right hind paw with 20 μL of 2% formalin solution diluted in saline. The total time spent licking the injected paw was recorded and separated into the early (0–5 min) and late (10–30 min) phases following formalin administration. The antinociceptive efficacy of the test samples was assessed by calculating the percentage inhibition of paw‐licking time.

(5) % inhibition of paw−licking time=Licking time of the control group−Licking time of the treatment groupLicking time of the control group×100.

### 2.7. In Silico Study

#### 2.7.1. Preparation of Protein and Ligand Structure

The COX‐2 protein structure was used for docking studies. Amino acid sequences were retrieved from the UniProt database. The crystal structure of COX‐2 was retrieved from the Protein Data Bank (PDB ID: 5 F1A) [25]. The protein structure was refined using BIOVIA Discovery Studio (version 21.1.0, Dassault Systèmes, France) to remove water molecules, ligands, other heteroatoms, and hydrogen atoms. Energy minimization was performed using SwissPDB Viewer (version 4.1.0, Swiss Institute of Bioinformatics (SIB), Basel, Switzerland). The ligand structure of PN‐H was retrieved from the PubChem database and converted into 3D format using Chem3D software.

#### 2.7.2. Docking Protocols

Molecular docking of the ligands with COX‐2 was conducted using PyRx (version 0.8.0.0, The Scripps Research Institute, La Jolla, CA, United States) [26]. Docking parameters were optimized, and ligand–protein interactions were visualized using PyMOL (version 2.5.5, Schrödinger Inc., New York, NY, United States) and BIOVIA Discovery Studio [27–29]. Ligands were docked against prepared protein structures, and the resulting complexes were analyzed to determine binding energies, identify potential binding sites, and characterize key amino acid interactions. These studies provided detailed insights into the binding modes and affinities for the target protein.

#### 2.7.3. ADME Analysis

SwissADME (https://www.swissadme.ch/index.php) and pkCSM‐pharmacokinetics (https://biosig.unimelb.edu.au/pkcsm/prediction) were used to predict the pharmacokinetic properties of the identified compounds. To be considered as an orally active medication, a compound must satisfy drug‐likeness criteria, commonly assessed by “Lipinski’s Rule of Five” [30, 31]. This rule evaluates molecular weight, lipophilicity (LogP), hydrogen‐bond donors, and hydrogen‐bond acceptors and donors to estimate a compound’s potential as an effective oral drug.

#### 2.7.4. Toxicity Prediction

The Prediction of Toxicity of Chemicals (ProTox‐II) platform (https://tox.charite.de/protox3/index.php?site=compound_input) was used to predict the potential toxicological profile of PN‐H. This computational tool is tailored for the early‐stage prediction of compound toxicity during drug development. For analysis, the molecular structure of PN‐H was converted into SMILES format and submitted to ProTox‐II. The platform predicts toxicity based on LD_50_‐related similarity and toxicophoric alerts, providing classifications and endpoints including hepatotoxicity, carcinogenicity, immunotoxicity, mutagenicity, and cytotoxicity [32, 33].

#### 2.7.5. Computational Forecasting of Bioactivity Profiles for Compounds: PASS Investigation via PASS Online

The Prediction of Activity Spectra for Substances (PASS) online tool was used to predict the potential biological activities of PN‐H based on its chemical structure. The analysis provided probability estimates for different biological activities, including anti‐inflammatory activity, based on the Pa (probability of activity) and Pi (probability of inactivity) values [34].

### 2.8. Statistical Analysis

All the experiments were performed in triplicate, whereas animal experiments included five animals per group. The experimental data were analyzed using GraphPad Prism (version 8.0). Results are reported as mean ± SD. One‐way analysis of variance (ANOVA) followed by Dunnett’s multiple‐comparison post hoc test was used to evaluate significance between the test samples. For the carrageenan‐induced paw edema assay, data were analyzed by two‐way repeated‐measures ANOVA with treatment and time as factors, followed by an appropriate multiple‐comparison test. A *p* value less than 0.05 was considered statistically significant.

## 3. Results

### 3.1. Characterization of Compound From CW‐EAF

PN‐H (Figure 3A), a guaiane‐type sesquiterpene, was identified using ^1^H, ^13^C, and 2D NMR spectroscopy together with LC‐MS analysis and comparison with published reference data (Table 1; Supporting Figures S2–S8). The observed molecular ion peak, together with the mass accuracy below 5 ppm, supported the proposed molecular formula and identity of the isolated compound. The structural assignment was further corroborated by comprehensive NMR spectroscopic data and comparison with published reference data. Although a quantitative chromatographic purity assessment was not available, the combined LC‐MS and NMR data provided strong evidence supporting the identity and structural characterization of PN‐H. To the best of our knowledge, this is the first report of PN‐H from *Curcuma wilcockii* rhizomes. PN‐H was previously reported as a constituent of *Curcuma phaeocaulis*, where phaeocaulisin‐type sesquiterpenes were isolated and evaluated for inhibitory effects on LPS‐induced nitric oxide production [7]. The present study therefore extends the occurrence of PN‐H to *C. wilcockii* and provides a more comprehensive evaluation of its anti‐inflammatory and antinociceptive properties using complementary in vitro, in vivo, and in silico approaches.

**FIGURE 3:**
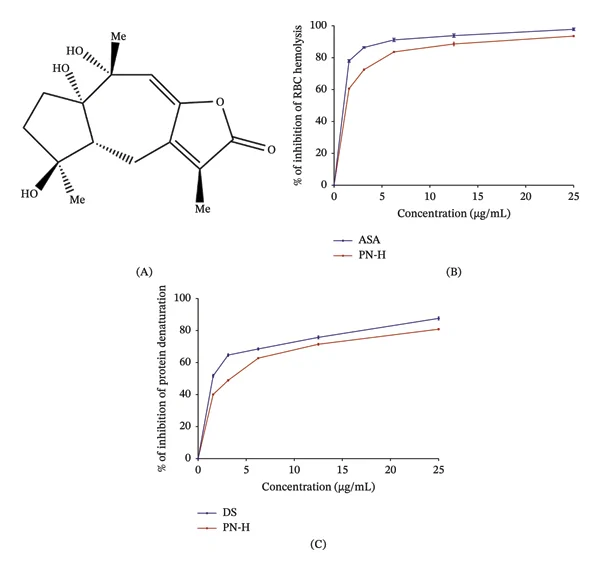
(A) Chemical structure of PN‐H. (B) Inhibition of heat‐induced red blood cell (RBC) hemolysis by PN‐H. Values are represented as mean ± SD, *n* = 3. (C) Inhibition of protein denaturation using egg albumin by PN‐H. Values are represented as mean ± SD, *n* = 3. Here, ASA: acetylsalicylic acid; DS: diclofenac sodium.

**TABLE 1:** Comparison of the NMR data of PN‐H with the reference.

Position	Reference data		PN‐H	
	*δ*_c_	*δ*_H_	*δ*_c_	*δ*_H_
1	83.5		83.3	

2	35.8	1.57, m	35.2	1.54, m
		2.45, m		2.49, m

3	41.4	1.59, m	41.0	1.69, m
		1.80, m		1.78, m

4	80.5		80.5	

5	51.8	2.06, dd (11.2, 4.9)	50.3	2.44, dd (12.2, 2.4)

6	22.3	2.69, m	22.1	2.72, m

7	151.7		151.9	

8	148.2		149.5	

9	120.0	5.51, m	118.6	5.56, m

10	77.7		75.6	

11	127.2		127.0	

12	172.3		172.3	

13	8.7	1.86, s	8.5	1.90, s

14	22.8	1.26, s	22.9	1.28, s

15	25.2	1.40, s	25.4	1.43, s

*Note:* PN‐H = Phaeocaulisin H.

### 3.2. In Vitro Anti‐Inflammatory Activity

#### 3.2.1. Heat‐Induced Hemolysis

The in vitro anti‐inflammatory activity test results are presented in Figure 3B, which was assessed using the human RBC membrane stabilization assay. The anti‐inflammatory response of the PN‐H was concentration‐dependent, with the percentage of protection increasing alongside sample concentration. At a concentration of 25 μg/mL, PN‐H exhibited 93.55% ± 0.42 inhibition of RBC hemolysis, comparable to the 97.83% ± 0.79 inhibition observed with standard ASA.

#### 3.2.2. Protein Denaturation Using Egg Albumin

The anti‐inflammatory activity of PN‐H was evaluated using the egg albumin denaturation assay. PN‐H was tested at multiple concentrations and compared with the standard drug. As shown in Figure 3C, PN‐H exhibits a significant dose‐dependent anti‐inflammatory effect. At 25 μg/mL, PN‐H achieves 80.86% ± 0.49 inhibition of protein denaturation compared with 87.62% ± 1.01 for DS, demonstrating its strong anti‐inflammatory potential.

### 3.3. In Vivo Anti‐Inflammatory Activity

#### 3.3.1. Carrageenan‐Induced Paw Edema

As shown in Figure 4, PN‐H at 20 mg/kg b.w. reduced (*p* < 0.0001) carrageenan‐induced paw edema by 50% ± 2.8 at 4 h post‐administration, whereas the 10 mg/kg b.w. dose inhibited (*p* < 0.0001) edema by 32.61% ± 2.29. At the same time point, the reference drug DS (10 mg/kg b.w.) produced a 54.35% ± 3.17 reduction (*p* < 0.0001) in paw edema.

**FIGURE 4:**
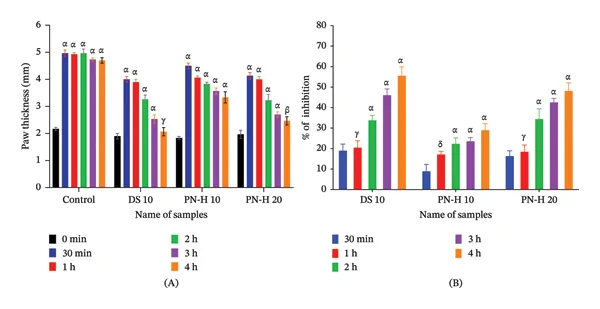
(A) Effect of PN‐H and DS on carrageenan‐induced paw edema test. Significant values are  ^α^ *p* < 0.0001,  ^β^ *p* < 0.001,  ^γ^ *p* > 0.05 compared to the control group. (B) % of inhibition of carrageenan‐induced paw edema by PN‐H. Significant values are  ^α^ *p* < 0.0001,  ^δ^ *p* < 0.01,  ^γ^ *p* > 0.05 compared to the DS 10 group. Here, DS 10: diclofenac sodium, 10 mg/kg b.w.; PN‐H 10: phaeocaulisin H 10 mg/kg b.w.; PN‐H 20: phaeocaulisin H 20 mg/kg b.w.

#### 3.3.2. Acetic Acid–Induced Abdominal Writhing Test

Oral administration of PN‐H at both tested doses significantly reduced abdominal writhing in mice in a dose‐dependent manner (*p* < 0.0001), at 10 and 20 mg/kg b.w. doses, the PN‐H inhibited the writhing response by 50.93% ± 0.82 and 79.13% ± 3.27, respectively, whereas the standard group demonstrated a 69.55% ± 1.63 inhibition with the DS (10 mg/kg b.w.) (Figure 5).

**FIGURE 5:**
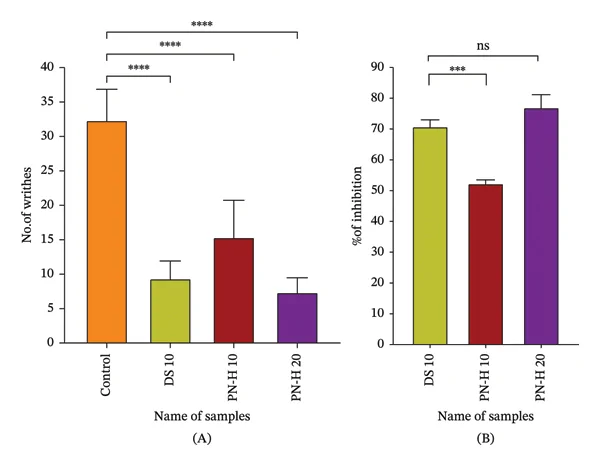
(A) Effect of PN‐H on acetic acid‐induced writhing responses in mice (*n* = 5). ^*∗∗∗∗*^ *p* < 0.0001, compared to the control group. (B) % of inhibition of the PN‐H using acetic acid‐induced writhing responses (*n* = 5). ^∗∗∗^ *p* < 0.001, ns = not significant, compared to the diclofenac sodium (DS) group. Here, DS 10: diclofenac sodium, 10 mg/kg b.w.; PN‐H 10: phaeocaulisin H 10 mg/kg b.w.; PN‐H 20: phaeocaulisin H 20 mg/kg b.w.

#### 3.3.3. Formalin‐Induced Licking Test

The biphasic formalin‐induced pain model was employed to further assess the antinociceptive activity of PN‐H, with results presented in Figure 6. PN‐H significantly reduced nociceptive behavior (*p* < 0.001) in both the early (neurogenic) and late (inflammatory) phases. In the early phase, PN‐H reduced pain responses by 56.77% ± 0.82 and 70.32% ± 1.63 at doses of 10 and 20 mg/kg b.w., respectively, compared to 69.68% ± 2.45 inhibition by DS. In the late phase, PN‐H produced 52.78% ± 1.63 and 77.78% ± 2.45 inhibition, while DS showed 75% ± 0.82 inhibition at 10 mg/kg b.w., indicating comparable efficacy of PN‐H at higher doses in inflammatory pain responses [35].

**FIGURE 6:**
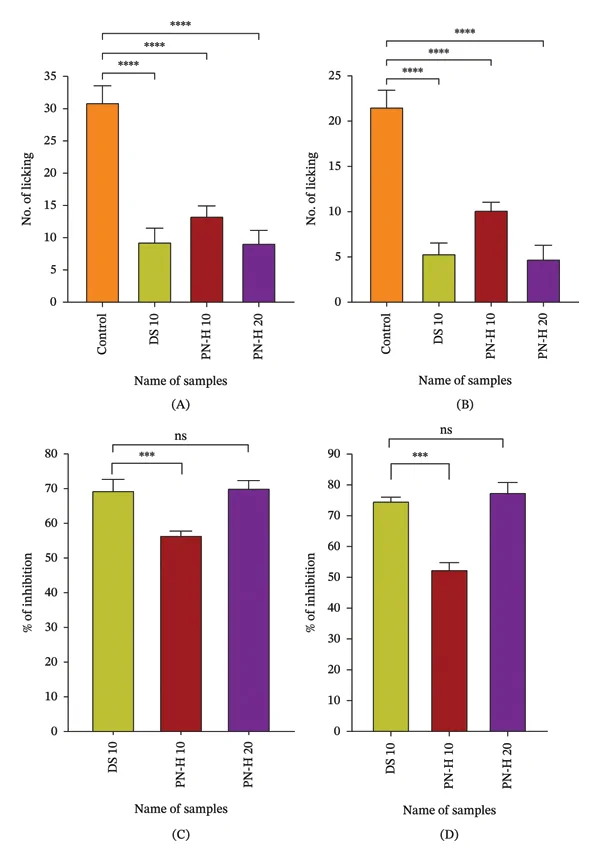
(A) Effect of PN‐H on paw licking in early phase, (B) Effect of PN‐H on paw licking in delayed phase after intraplantar injection of formalin in the formalin‐induced licking test. Values represent mean ± SD for each group (*n* = 5). ^∗∗∗^ *p* < 0.001 compared to the control group. (C) % inhibition of PN‐H by formalin (early phase), (D) % inhibition graph of PN‐H by formalin (delay phase). Values represent mean ± SD for each group (*n* = 5). ^∗∗∗^ *p* < 0.001, ns = not significant, compared to the diclofenac sodium (DS) group. Here, DS 10: diclofenac sodium, 10 mg/kg b.w.; PN‐H 10: phaeocaulisin H 10 mg/kg b.w.; PN‐H 20: phaeocaulisin H 20 mg/kg b.w.

### 3.4. In Silico Study

#### 3.4.1. Molecular Docking

The COX‐2 crystal structure (PDB ID: 5 F1A) was used for the docking study. The docking study identified PN‐H with a binding energy of −8.4 kcal/mol, surpassing that of DS (−7.7 kcal/mol), indicating a strong affinity for the COX‐2. From PubChem, we extracted chemical names using Canonical SMILES. The binding interactions between PN‐H and COX‐2 were visualized and shown in Figure 7. In the 5 F1A protein, PN‐H showed seven conventional hydrogen bonds with GLN A:241, ARG A:333, GLU A:236, ASP A:229, with bond distances ranging from 2.04797 to 2.61212 Å. Additionally, PN‐H formed one pi‐donor hydrogen bond with SER B:143, at a bond distance of 3.08422 Å. Moreover, PN‐H also showed that two hydrophobic (alkyl) interactions were observed with LEU A:224 and LEU A: 238 and the range of bond distance lies between 4.20261 and 5.11738 Å. The ligand also established one hydrophobic (pi‐alkyl) interaction with TRP A:139, at a bond distance of 5.06722 Å.

**FIGURE 7:**
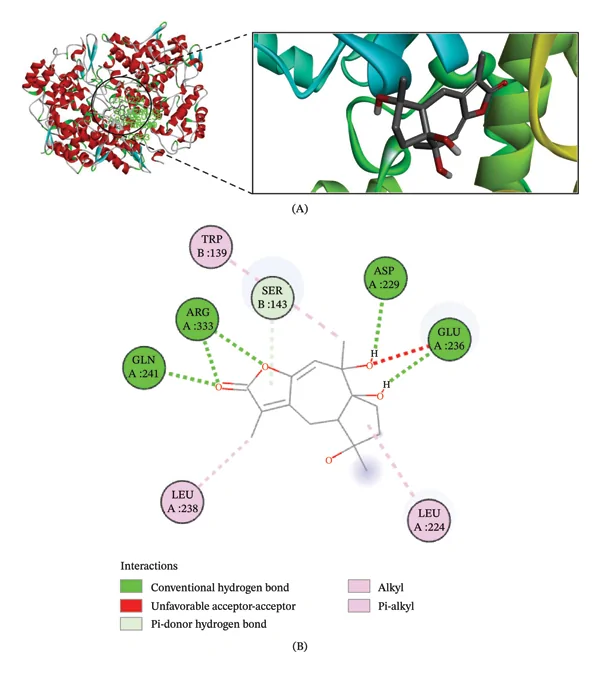
Molecular docking interaction of the compound PN‐H with the target protein (COX‐2). (A) 3D binding conformation of the ligand within the active site of the protein. (B) 2D interaction diagram illustrating hydrogen bonding and hydrophobic interactions between the ligand and amino acid residues in the binding pocket. In the 2D diagram, atom colors are represented as follows: carbon (grey), oxygen (red), nitrogen (blue), and hydrogen (white). Hydrogen bonds are indicated by dashed green lines, while hydrophobic interactions are represented by light pink arcs.

#### 3.4.2. ADME Properties

Pharmacokinetic profiling is a critical step in drug development, providing insights into absorption, distribution, metabolism, and excretion (ADME), as well as the potential toxic or therapeutic effects of a compound. Many drug candidates fail in clinical trials due to inadequate pharmacokinetic properties. Therefore, computational approaches are widely applied to predict ADME and drug‐likeness parameters at an early stage. The canonical SMILES of PN‐H was analyzed using the SwissADME platform. SwissADME generated a radar plot (Figure 8), in which each axis represents a key physicochemical or pharmacokinetic parameter, and the shaded pink region delineates the optimal range for drug‐like behavior. PN‐H was largely located within the recommended physicochemical space represented in the SwissADME radar plot, supporting its predicted drug‐like properties.

**FIGURE 8:**
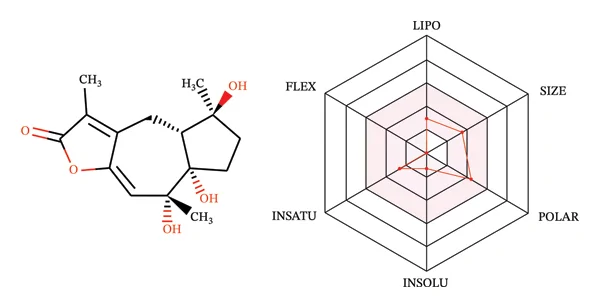
SwissADME bioavailability radar of PN‐H, where the pink areas represent each property (lipophilicity, molecular weight, solubility, and flexibility).

The predicted ADMET properties of PN‐H were compared with the reference drug DS. PN‐H demonstrated favorable pharmacokinetic characteristics in several parameters, including blood–brain barrier (BBB) permeability and non‐hepatotoxicity. However, the predicted human intestinal absorption (HIA) for PN‐H was relatively lower (38.9%) compared with DS (77.7%), indicating comparatively limited intestinal permeability. Despite this difference, other pharmacokinetic parameters suggested acceptable drug‐like properties for PN‐H, summarized in Table 2.

**TABLE 2:** Physicochemical properties and ADMET profiles of PN‐H and standard DS.

Properties			PN‐H	DS
Molecular properties		PubChem CID	71725779	5018304
		Molecular weight g/mol	280.32	318.13
		Num. H‐bond acceptors	5	2
		Num. H‐bond donors	3	1
		Fraction Csp3	0.67	0.07
		TPSA (Å^2^)	86.99	52.16
		Log Po/w (iLOGP)	1.92	−11.74
		Log S (ESOL)	−1.33	−4.76
		Lipinski rule	Yes; 0 violation	Yes; 0 violation
		Bioavailability score	0.55	0.55

ADMET	Absorption	Water solubility (log mol/L)	−2.858	−3.405
		Human intestinal absorption (%)	38.885	77.738
	Distribution	BBB permeability (log BB)	−0.568	0.074
		CNS permeability (log PS)	−3.439	−2.751
	Metabolism	CYP3A4 substrate	No	No
		CYP3A4 inhibitor	No	No
	Excretion	Total clearance (log mL/min/kg)	1.029	−0.83
		Renal OCT2 substrate	No	No
	Toxicity	Human max. tolerated dose (log mg/kg/day)	0.753	0.681
		Oral rat acute toxicity, LD_50_ (mol/kg)	2.087	2.162

*Note:* PN‐H = Phaeocaulisin.

Abbreviation: DS, Diclofenac sodium.

#### 3.4.3. Toxicity Predictions

ProTox‐II was used to predict the toxicological profile of PN‐H and DS (Table 3). PN‐H showed an LD_50_ of 130 mg/kg (Class 3) and was inactive for hepatotoxicity, carcinogenicity, mutagenicity, and cytotoxicity. In comparison, DS exhibited a predicted LD_50_ of 53 mg/kg (Class 3) and was predicted to be hepatotoxic. These findings provide preliminary computational information on the potential toxicity profiles of PN‐H and DS and support further experimental evaluation of PN‐H.

**TABLE 3:** In silico toxicity prediction of PN‐H with standard DS.

Properties	PN‐H	DS
LD_50_ (mg/Kg)	130 mg/kg (Class 3)	53 mg/kg (Class 3)
Hepatotoxicity	Inactive	Active
Carcinogenicity	Inactive	Inactive
Immunotoxicity	Active	Inactive
Mutagenicity	Inactive	Inactive
Cytotoxicity	Inactive	Inactive

*Note:* PN‐H = phaeocaulisin H, LD_50_ = median lethal dose.

Abbreviation: DS, diclofenac sodium.

#### 3.4.4. In Silico PASS Prediction Study

PASS analysis was performed to estimate the potential biological activities of PN‐H based on its chemical structure. The analysis predicted multiple potential biological activities for PN‐H, with Pa values greater than Pi for all activities presented in Table 4. Among these anti‐inflammatory activities showed a Pa value of 0.383 and a Pi value of 0.105, indicating a predicted probability of activity greater than inactivity. These findings represent computational predictions and should not be interpreted as experimental confirmation of the predicted biological activities.

**TABLE 4:** Predicted bioactivities of PN‐H based on in silico PASS analysis.

Pa	Pi	Activity
0.382	0.054	Antifungal
0.240	0.063	Anthelmintic
0.264	0.146	Anthelmintic (Nematodes)
0.374	0.042	Antihypercholesterolemic
0.139	0.077	Antihyperlipoproteinemic
0.233	0.173	Anti‐infective
0.383	0.105	Anti‐inflammatory
0.282	0.109	Anti‐inflammatory, ophthalmic
0.153	0.105	Antileukemia
0.215	0.141	Antimetastatic
0.041	0.026	Antimitotic, podophyllotoxin‐like
0.183	0.085	Antimutagenic

Abbreviations: Pa, Probability of activity; Pi, Probability of inactivity.

## 4. Discussion

Current anti‐inflammatory and analgesic drugs, including NSAIDs, corticosteroids, and opioids, may cause adverse effects with prolonged use, highlighting the need for safer therapeutic alternatives [36]. Medicinal plants remain important sources of anti‐inflammatory and analgesic compounds, including flavonoids, alkaloids, terpenoids, and phenolic acids, which can modulate inflammatory pathways through mechanisms involving COX inhibition, cytokine regulation, and antioxidant activity [37, 38]. Among medicinal plants, the genus *Curcuma* has received considerable attention for its pharmacological diversity, with species such as *C. longa*, *C. zedoaria*, and *C. phaeocaulis* and their bioactive constituents, including curcumin, demethoxycurcumin, and zedoarondiol, showing anti‐inflammatory and analgesic effects [39]. Recent mechanistic studies of isolated sesquiterpenes have further demonstrated that these compounds can modulate key inflammatory mediators, including COX‐2, iNOS, TNF‐α, and IL‐6, partly through regulation of NF‐κB and related signaling pathways [40].

The identification of PN‐H in *C. wilcockii* represents a notable phytochemical finding, as PN‐H and other phaeocaulisin‐type sesquiterpenes were previously reported from *C. phaeocaulis* [7]. In the present study, PN‐H demonstrated significant anti‐inflammatory and antinociceptive activities across complementary in vitro and in viv*o* models. The occurrence of PN‐H in both *C. phaeocaulis* and *C. wilcockii* expands the reported distribution of this compound within the genus *Curcuma*. Further phytochemical and biosynthetic studies are needed to determine whether the occurrence of PN‐H in these species has any chemotaxonomic significance.

PN‐H significantly inhibited heat‐induced hemolysis, indicating membrane‐stabilizing activity. Because membrane stabilization may limit the release of lysosomal contents and subsequent inflammatory responses, this effect may contribute to the observed anti‐inflammatory activity of PN‐H [41, 42]. PN‐H also showed strong inhibition of protein denaturation, further supporting its anti‐inflammatory potential. Its activity was comparable to that of DS, suggesting that membrane stabilization and inhibition of protein denaturation may contribute to its observed activity [41].

The in vivo findings further supported anti‐inflammatory activity of PN‐H. In the carrageenan‐induced paw edema assay, PN‐H significantly reduced paw edema, with the greatest inhibition observed during the later phase of the response (2–4 h). Because this phase is largely associated with prostaglandin‐mediated inflammation [43], the observed effect suggests that PN‐H may modulate prostaglandin‐associated inflammatory signaling [44].

In the acetic acid‐induced writhing test, PN‐H significantly reduced the number of writhes, indicating peripheral antinociceptive activity, potentially through inhibition of prostaglandin‐mediated signaling. In the formalin test, both the early (neurogenic) and late (inflammatory) phases of nociception were significantly suppressed by PN‐H. The inhibition of the early phase may suggest involvement of neurogenic or central pathways, whereas suppression of the late phase is consistent with peripheral anti‐inflammatory activity [35, 45]. However, the potential central component remains preliminary and requires further mechanistic validation.

Compared with well‐characterized *Curcuma* compounds such as curcumin and zedoarondiol, PN‐H appears to exhibit a distinct anti‐inflammatory profile. Curcumin primarily targets NF‐κB signaling although its inhibition of NF‐κB DNA binding in LPS‐stimulated RAW264.7 macrophages occurs at relatively high concentrations (IC_50_ > 50 μM) [46]. In contrast, PN‐H showed a favorable predicted interaction with COX‐2, while zedoarondiol has been reported to inhibit iNOS and COX‐2 and suppress NO, PGE_2_, TNF‐α, IL‐6, and IL‐1β production in the same model [47]. Similarly, isolinderalactone has been reported to suppress COX‐2 and iNOS expression and reduce PGE_2_ and pro‐inflammatory cytokines, partly through inhibition of NF‐κB signaling [40]. Arnicolide B and arnicolide C have also been shown to suppress NO and PGE_2_ production and downregulate iNOS and COX‐2, accompanied by reduced TNF‐α and IL‐6 production [48]. These findings support the potential involvement of multiple inflammatory mediators and signaling pathways in the anti‐inflammatory effects of isolated sesquiterpenes. In the present study, the favorable predicted interaction of PN‐H with COX‐2 provides a potential mechanistic basis for its observed activity, although this prediction requires experimental validation.

The potential pharmacological profile of PN‐H was evaluated using PASS, which predicted a high likelihood of anti‐inflammatory activity, consistent with the effects observed in our in vitro and in vivo models [34]. To further explore its possible molecular basis, molecular docking was performed against COX‐2 enzyme, a key mediator of inflammation. PN‐H showed favorable binding affinity and formed conventional and *π*‐donor hydrogen bonds, as well as alkyl and *π*‐alkyl interactions. These interactions suggest that COX‐2 may be a potential molecular target associated with the observed anti‐inflammatory activity of PN‐H. This interpretation is consistent with previous studies of isolated sesquiterpenes reporting suppression of COX‐2 and iNOS expression and reduced production of pro‐inflammatory cytokines [49]. However, the docking findings remain predictive and require experimental validation, such as assessment of COX‐2 and inflammatory cytokines by Western blotting or ELISA to confirm pathway involvement.

Pharmacokinetic assessment using SwissADME indicated a favorable drug‐like profile of PN‐H. It showed compliance with Lipinski’s Rule of Five and suitable physicochemical properties, including molecular weight, lipophilicity, and hydrogen‐bonding capacity [50]. The SwissADME radar plot further demonstrated that PN‐H largely fell within the optimal range for orally administered drugs, supporting its potential for systemic administration. Interestingly, PN‐H was predicted to exhibit low BBB permeability, suggesting limited CNS exposure and potentially reducing the likelihood of CNS‐related adverse effects. However, these in silico predictions require experimental validation [51].

Despite favorable ADME characteristics, the relatively low predicted HIA (38.9%) of PN‐H may limit its oral bioavailability. This limitation could potentially be addressed through structural optimization or prodrug strategies. For example, conversion of tenofovir to the prodrug tenofovir disoproxil fumarate has been reported to improve its oral bioavailability [52].

In silico toxicity predictions provided preliminary insights into the potential safety profile. PN‐H had a predicted LD_50_ of 130 mg/kg (Class 3) and was predicted to be non‐hepatotoxic, non‐carcinogenic, non‐mutagenic, and non‐cytotoxic, whereas DS was predicted to be hepatotoxic. PN‐H was also well tolerated during the in vivo anti‐inflammatory experiments, with no observable adverse effects at the tested doses. However, the toxicity assessment in the present study was entirely computational, and no experimental acute or subacute toxicity studies were performed. Further acute and subacute toxicity studies are warranted to establish the safety profile of PN‐H more comprehensively [32].

The integrated pharmacological, pharmacokinetic, and in silico analyses further support the potential of PN‐H as a bioactive constituent of *C. wilcockii*.

Collectively, these findings identify PN‐H as a promising natural compound with notable anti‐inflammatory and analgesic activities, supported by complementary experimental and computational evidence. The integrated pharmacological, pharmacokinetic, and in silico analyses further highlight the potential of PN‐H as a bioactive constituent of *C. wilcockii*. Although the present findings provide a basis for further investigation of the biological activity of PN‐H, further studies are needed to clarify its molecular mechanisms and to assess its efficacy in chronic inflammatory models. Isolation of PN‐H in sufficient quantities will facilitate these investigations and enable further pharmacological optimization. Overall, the findings support PN‐H as a promising lead for further investigation in the development of novel plant‐derived anti‐inflammatory agents.

### 4.1. Proposed Mechanism of Action

Carrageenan‐induced inflammation is known to involve mast cell activation, leading to the release of histamine, a pivotal mediator that promotes the liberation of arachidonic acid from membrane phospholipids [53]. Similarly, irritant agents such as formalin or acetic acid stimulate mast cell degranulation and histamine release [54]. The released histamine subsequently activates phospholipase A2, which hydrolyzes membrane phospholipids to liberate arachidonic acid. This arachidonic acid subsequently metabolized through the cyclooxygenase pathway, generating prostaglandin G2 and prostaglandin H2, which serve as precursors for several inflammatory mediators, including prostacyclin and thromboxane. These mediators contribute to increased vascular permeability, pain, and edema during the inflammatory response [55, 56]. Based on these established inflammatory processes, the findings suggest that the anti‐inflammatory activity of PN‐H may involve modulation of prostaglandin‐related signaling. Molecular docking demonstrated favorable interactions of PN‐H within the COX‐2 binding site, supporting a potential role of COX‐2‐associated signaling in its activity.

## 5. Conclusion

Phaeocaulisin H (PN‐H), isolated from the rhizomes of *C. wilcockii*, exhibited significant anti‐inflammatory activity in both in vitro and in vivo models. Its therapeutic potential was further supported by molecular docking analyses, which revealed favorable interactions with the COX‐2 binding site and suggested a possible mechanistic basis for its anti‐inflammatory effects. The occurrence of PN‐H in *C. wilcockii* expands the known distribution of this rare guaiane‐type sesquiterpene and contributes to the growing understanding of phytochemical diversity within the genus *Curcuma*. Collectively, these findings highlight the pharmacological potential of PN‐H and support further investigation of PN‐H as a potential natural anti‐inflammatory candidate. The potential involvement of COX‐2‐associated signaling warrants further experimental validation.

## 6. Limitations of the Study

This study provides a comprehensive preliminary evaluation of PN‐H. Although PN‐H demonstrated significant anti‐inflammatory and antinociceptive effects and in silico docking supported its interaction with COX‐2, experimental molecular assays (e.g., Western blot or ELISA for COX‐2, TNF‐α, and IL‐6) were not performed to confirm the precise pathway involvement. Moreover, direct COX‐2 inhibition was not assessed, and therefore the potential involvement of COX‐2‐associated signaling requires further biochemical validation. Furthermore, antinociceptive activity was evaluated using only acetic acid‐induced writhing and formalin tests, which provide complementary evidence of nociceptive activity but do not fully characterize the underlying analgesic mechanisms. Additionally, the study was limited to acute models and did not evaluate chronic inflammation or long‐term safety. Future investigations employing molecular assays, complementary nociceptive models, and advanced in vivo approaches are necessary to validate the mechanistic pathways and confirm the translational potential of PN‐H.

**Nomenclature**
ADMET: Absorption, distribution, metabolism, excretion, and toxicity
ASA: Acetylsalicylic acid
C. wilcockii: Curcuma wilcockii
CME: Crude methanol extract
b.w.: Body weight
COX‐2: Cyclooxygenase‐2
DS: Diclofenac sodium
EAF: Ethyl acetate fraction
CHF: Chloroform fraction
HRBC: Heat‐induced red blood cell
ICDDRB: International Centre for Diarrheal Disease Research Bangladesh
LD_50_
: Median lethal dose
NHF: n‐Hexane fraction
NSAIDs: Nonsteroidal anti‐inflammatory drugs
PASS: Prediction of activity spectra for substances
PDB: Proteins Data Bank
PN‐H: Phaeocaulisin H
CW‐EAF: *Curcuma wilcockii* ethyl acetate fraction

## Author Contributions

A. B. M. Ashraful: conceptualization, data curation, formal analysis, methodology, software, and writing–original draft. Md. Sabbir Hossain: formal analysis, methodology, and writing–review and editing. Farhad Hossain: formal analysis and software. Joy Sarker: software and writing–original draft. Md. Najem Uddin: formal analysis and resources. Aziz Abdur Rahman, Md. Golam Sadik, and Mamunur Rashid: validation and writing–review and editing. A. H. M. Khurshid Alam: investigation, resources, supervision, validation, visualization, and writing–review and editing. All authors reviewed the manuscript carefully.

## Funding

This research did not receive any specific grant from funding agencies in the public, commercial, or not‐for‐profit sectors.

## Ethics Statement

All animal experiments and human blood collection procedures were conducted in accordance with the relevant institutional ethical guidelines and regulations. The animal experiments were approved by the Institute of Biological Sciences (IBSc), Rajshahi University, Bangladesh (license no. 72 (23)/320/IAMEBBC/IBSc), while the human blood collection for the HRBC membrane stabilization assay received separate ethical approval from the IBSc, Rajshahi University, Bangladesh (approval no. 218/320(41)/IAMEBBC/IBSc). Written informed consent was obtained from the healthy volunteer prior to blood collection. The relevant ethical approval and informed consent documentation are provided in the Supporting File.

## Conflicts of Interest

The authors declare no conflicts of interest.

## Supporting Information

Additional supporting information can be found online in the Supporting Information section.

## Supporting information


**Supporting Information** The supporting informations provide additional data and supporting information that complement the findings of the present study. Detailed supporting data are presented in Supporting Tables S1–S2, while supporting Figures S1–S8 illustrate additional results and relevant analyses. These materials provide further support for the findings discussed in the main text.

## Data Availability

All data generated or analyzed during this study are included in this article and its supporting information files.
