# Supplementary material for: Anti‐Inflammatory and Antinociceptive Potential of Phaeocaulisin H From Curcuma wilcockii Rhizomes (Zingiberaceae) in In Vitro, In Vivo, and In Silico Studies
Source: Adv Pharmacol Pharm Sci. 2026 Nov 9;2026:2384820. doi: 10.1155/adpp/2384820 (PMC13748800; doi:10.1155/adpp/2384820)
Supplement: Supplementary file 1 [file ADPP-2026-2384820-s001.docx]

**Supplementary File**

**Anti-inflammatory and antinociceptive potential of phaeocaulisin H from *Curcuma wilcockii* rhizomes (Zingiberaceae) in *in vitro*, *in vivo*, and *in silico* studies**

A. B. M. Ashraful^1^, E-mail: abmashraful98@gmail.com, ORCID: https://orcid.org/0009-0004-2264-4488; Md. Sabbir Hossain^1,2^, E-mail: hossainsabbir6152@gmail.com; Farhad Hossain ^1,3^, E-mail: fhayon07@gmail.com, Joy Sarker^1^, E-mail: joyru.ac.bd@gmail.com; Md. Najem Uddin^4^, E-mail: najem.ru@bcsir.gov.bd; Aziz Abdur Rahman^1^, E-mail: aziz2002@ru.ac.bd; Md. Golam Sadik^1^, E-mail: gsadik2@yahoo.com; Mamunur Rashid^1^, E-mail: mamun69jp@yahoo.com; AHM Khurshid Alam^1,^*, E-mail: [khurshid_alam@ru.ac.bd](mailto:khurshid_alam@ru.ac.bd)

^1^Department of Pharmacy, University of Rajshahi, Rajshahi-6205, Bangladesh.

^2^Department of Pharmacy, Pabna University of Science and Technology, Pabna-6600, Bangladesh.

^3^Department of Pharmacy, State University of Bangladesh, Dhaka-1461, Bangladesh.

^4^Bangladesh Council of Scientific and Industrial Research (BCSIR), Dhanmondi, Dhaka 1205, Bangladesh.

***Correspondence**: Dr. AHM Khurshid Alam, Professor, Department of Pharmacy, University of Rajshahi, Rajshahi-6205, Bangladesh. Email: [khurshid_alam@ru.ac.bd](mailto:khurshid_alam@ru.ac.bd). Phone Number: +8801719634499, Fax: +880721750064.

**Table S1:** Several fractions with their corresponding test tube and amount of solvent used with their ratio of EAF after doing column chromatography.

| **Fraction numbers** | **Test tubes** | **Solvent systems** | **Amount of solvent** |
| --- | --- | --- | --- |
| Fr-1 | 1-50 | 100% CH | 350ml |
|  |  | CH+MH – 99.75+0.25 | 200ml |
|  |  | CH+MH – 99.5+0.5 | 150ml |
|  |  | CH+MH – 99.25+0.75 | 150ml |
| Fr-2 | 51-74 | CH+MH – 99+1 | 200ml |
| Fr-3 | 75-90 | CH+MH – 98.75+1.25 | 250ml |
| Fr-4 | 91-127 | CH+MH – 98.5+1.5 | 800 ml |
| Fr-5 | 128-159 |  |  |
| Fr-6 | 160-180 |  |  |
| Fr-7 | 181-202 | CH+MH – 98+2  CH+MH – 97.5+2.5  CH+MH – 95+5  CH+MH – 90+10 | 100ml  100ml  100ml  100ml |
| Fr-8 | 203-207 | CH+MH – 80+20 | 100ml |

| **Position** | **δ_c_** | **δ_H_** |
| --- | --- | --- |
| 1 | 83.3 |  |
| 2 | 35.2 | 1.54,m  2.49,m |
| 3 | 41.0 | 1.69,m  1.78,m |
| 4 | 80.5 |  |
| 5 | 50.3 | 2.44,dd (12.2, 2.4) |
| 6 | 22.1 | 2.72,m |
| 7 | 151.9 |  |
| 8 | 149.5 |  |
| 9 | 118.6 | 5.56,m |
| 10 | 75.6 |  |
| 11 | 127.0 |  |
| 12 | 172.3 |  |
| 13 | 8.5 | 1.90,s |
| 14 | 22.9 | 1.28,s |
| 15 | 25.4 | 1.43,s |

**Table S2:** ^1^H and ^13^C NMR data for compound CWA-EAF.

**Figure_S1:** Schematic representation of solvent-solvent partitioning of the CME of *C. wilcockii* rhizome

**Figure_S2:** ^1^H-NMR spectrum of CWA-EAF

**Figure_S3:** ^13^C-NMR spectrum of CWA-EAF

**Figure_S4:** DEPT-135 spectrum of CWA-EAF

**Figure_S5:** COSY of CWA-EAF

**Figure_S6:** HSQC of CWA-EAF

**Figure_S7:** HMBC of CWA-EAF

**Figure_S8:** LC-MS spectrum for pure compound
